# Dual-Purpose Materials Based on Carbon Xerogel Microspheres (CXMs) for Delayed Release of Cannabidiol (CBD) and Subsequent Aflatoxin Removal

**DOI:** 10.3390/molecules24183398

**Published:** 2019-09-19

**Authors:** Farid B. Cortés, Karol Zapata, Benjamín A. Rojano, Francisco Carrasco-Marín, Jaime Gallego, M. Alejandra Hernández, Camilo A. Franco

**Affiliations:** 1 Grupo de Investigación Fenómenos de Superficie—Michael Polanyi, Facultad de Minas, Universidad Nacional de Colombia, Sede Medellín, Medellín 050034, Colombia; kzapata@unal.edu.co (K.Z.); mahernandezmu@unal.edu.co (M.A.H.); caafrancoar@unal.edu.co (C.A.F.); 2 Grupo de Investigación Yacimientos de Hidrocarburos, Facultad de Minas, Universidad Nacional de Colombia, Sede Medellín, Medellín 050034, Colombia; 3 Grupo de Investigación Química de Los Productos Naturales y Los Alimentos, Facultad de Ciencias, Universidad Nacional de Colombia, Sede Medellín, Medellín 050034, Colombia; brojano@unal.edu.co; 4 Grupo de Investigación Materiales de Carbón, Departamento de Química Inorgánica, Facultad de Ciencias, Universidad de Granada, Granada 18071, Spain; fmarin@ugr.es; 5 Química de Recursos Energéticos y Medio Ambiente, Instituto de Química, Universidad de Antioquia UdeA, Medellín 050010, Colombia; andres.gallego@udea.edu.co

**Keywords:** adsorption, cannabidiol (CBD), aflatoxin, carbon xerogel microspheres (CXMs), delayed-release, removal, in vitro conditions

## Abstract

The main objective of this study is to develop a novel dual-purpose material based on carbon xerogel microspheres (CXMs) that permits the delayed release of cannabidiol (CBD) and the removal of aflatoxin. The CXMs were prepared by the sol-gel method and functionalized with phosphoric acid (CXMP) and melamine (CXMN). The support and the modified materials were characterized by scanning electronic microscopy (SEM), N_2_ adsorption at −196 °C, X-ray photoelectron spectroscopy (XPS), and zeta potential. For the loading of the cannabidiol (CBD) in the porous samples, batch–mode adsorption experiments at 25 °C were performed, varying the concentration of CBD. The desorption kinetics was performed at two conditions for simulating the gastric (pH of 2.1) and intestinal (pH of 7.4) conditions at 37 °C based on in vitro CBD release. Posteriorly, the samples obtained after desorption were used to study aflatoxin removal, which was evaluated through adsorption experiments at pH = 7.4 and 37 °C. The adsorption isotherms of CBD showed a type I(b) behavior, with the adsorbed uptake being higher for the support than for the modified materials with P and N. Meanwhile, the desorption kinetics of CBD at gastric conditions indicated release values lower than 8%, and the remaining amount was desorbed at pH = 7.4 in three hours until reaching 100% based on the in vitro experiments. The results for aflatoxin showed total removal in less than 30 min for all the materials evaluated. This study opens a broader landscape in which to develop dual-purpose materials for the delayed release of CBD, improving its bioavailability and allowing aflatoxin removal in gastric conditions.

## 1. Introduction

Humanity has used the cannabis plant for thousands of years in almost all cultures for medicinal, recreational, and ritualistic purposes [1]. The first approach to isolate the chemical compound synthesis and understand the chemistry of the cannabis plant was developed by Mechoulam’s research group [2]. The pharmaceutical field is one of the main areas of cannabinoid use due to its analgesic, hypotension, ocular, and antiemesis properties. In this sense, medications have been developed to treat neuropathic pains, traumas, cancer-related disorders, multiple sclerosis, nausea and vomiting induced by chemotherapy, Parkinson’s, Huntington’s disease, and Alzheimer’s disease, and current research is also focused on anorexia, dementia, Tourette’s syndrome, and fibromyalgia, among others [3].

Cannabinoids are substances that usually have a carbocyclic structure and are generally formed by three rings: cyclohexene, tetrahydropyran, and benzene [4]. The most important chemical compounds of the cannabinoids are tetrahydrocannabinol (THC), cannabidiol (CBD), and cannabinol (CBN) [5]. It is well known that THC and CBD are substances of therapeutic interest and that each of them has specific functions. Notwithstanding positive facts in the medical field, cannabinoids have psychoactive effects in the clinical applications studied. On the other hand, as CBD has a detached tetrahydrofuran ring it lacks psychoactive properties, making it an appropriate cannabinoid for the treatment of diseases [6,7]. However, due to its lipophilic nature, CBD has low oral adsorption, which implies a low bioavailability of approximately 9–13% [8]. In addition to this problem, the compound is susceptible to metabolism since the liver can eliminate up to 90% [9,10]. Hence, oil-based CBD capsules have been formulated for oral delivery in humans. However, due to its poor solubility, absorption in the gastrointestinal tract is low, obtaining only 6% of bioavailability due to the pass metabolism [11]. 

These limitations open a window for research to provide solutions to the issues of solubility and bioavailability of CBD compounds. In this sense, some technologies such as nanocarriers, encapsulated and active compounds (ACs) loaded onto porous materials, are suitable for CBD delivery, and could favor bioavailability and reduce the therapeutic dosage [12,13]. Thus, different authors have studied the applications of CBD in the medical field, and have focused on improving the oral bioavailability of this compound as well as on nanocarriers [10]. Aparicio-Blanco et al. [3] prepared monodisperse lipid nanocapsules (LNCs) as biocompatible and biodegradable materials modified and loaded with CBD for glioma therapy. The CBD was encapsulated onto LNCs to test in vitro efficacy, obtaining 20-nm and 50-nm nanoparticles. 

Cherniakov et al. [14] designed and developed pro-nanolipospheres (PNLs) to create a lipid-based formulation with the unique addition of a natural absorption enhancer (piperine). The PNLs upon contact with water self-emulsify, forming an Oil in Water (O/W) nano-emulsion. The resulting nanoparticles entrap the lipophilic drug in their core, achieving a solubilized state in an aqueous medium. Pharmacokinetic profiles were described through concentration plasmatic kinetics for 20 h, confirming that the nanometric size of the particles (30–50 nm) is of great importance since it enables penetration to the inter-villous spaces at the intestinal brush border, thus increasing the available surface area for absorption. Nakano et al. [15] developed a CBD nanoemulsion with particle size between 14 nm and 35 nm that signaled an improvement in CBD solubility and absorption. Concentration plasmatic kinetics and pharmacokinetic analyses showed that nanoemulsion could considerably recover the bioavailability. These nanoparticles improve efficiency substantially, entrapping water-soluble drugs. It is worth mentioning that these type of techniques can represent an alternative to the traditional methods used for CBD dosage (ointments, oral dosages, and patches, among others).

However, to the best of our knowledge, in the specialized literature there are no reported studies of CBD loaded onto organic porous materials for drug delivery in order to improve solubility and bioavailability. Also, these particles, after CBD release, can entrap polar toxins such as aflatoxin. The aflatoxin mycotoxins are a toxic secondary metabolite produced by fungi. When the conditions of humidity and temperature are adequate, fungi proliferate and form colonies that can produce high concentrations of toxins. Cereals, nuts, and fruits are the places for growth of fungi and the consequent production of mycotoxins. Exposure to high levels of mycotoxins causes acute necrosis, cirrhosis, and liver cancer. In the long term, these compounds have carcinogenic, mutagenic, teratogenic, estrogenic, immunotoxic, nephrotoxic, and neurotoxic effects.

Therefore, this study aims to develop a dual-purpose material based on carbon xerogel microspheres (CXMs) for loading with CBD as an active molecule at 25 °C, and to evaluate its release under in vitro conditions at 37 °C as well as the removal capacity for aflatoxin in intestinal conditions. The CXMs were synthesized based on the sol-gel method, and the surface was modified with phosphoric acid and melamine to alter the surface chemistry. The CBD load was carried out using batch–mode adsorption experiments at different concentrations of CBD and with a fixed amount of CXMs. Also, the desorption kinetics at in vitro conditions (gastric pH = 2.1 and intestinal pH = 7.4) were studied. After the CBD release, the materials were evaluated for the removal of the aflatoxin based on the adsorption kinetics in the intestinal conditions. 

## 2. Results and Discussion

### 2.1. Characterization of Carbon Xerogel Microspheres 

Carbon xerogel microspheres (CXMs) were obtained using the sol-gel method and were functionalized with melamine (CMXN) and phosphoric acid (CMXP) to modify the support surface. Table 1 summarizes the atomic concentration on the surface of CXM before and after functionalization as determined through X-ray photoelectron spectroscopy (XPS) high-resolution analyses, as well as the values obtained for the zeta potential at zero charges (pH_pzc_). The theorical concentration of heteroatoms (P and N) for the modification of the CXM was 3.0%, in mass fraction. However, the results obtained by XPS analysis showed that the phosphorous and nitrogen concentrations were 2.2% and 1.1% in the CXMP and CXMN samples, respectively, obtaining impregnation efficiencies of P and N of 70% and 30%. This is in agreement with the results reported by Bailón-García [16].

The point of zero charges (pH_pzc_) is a key parameter for determining the adsorbent affinity for a specific adsorbate. Table 1 shows that the pH_pzc_ CXMP is lower than the for CXM and CXMN samples; this is due to the use of an acid as a modifier (H_3_PO_4_) that promoted the crosslink with phosphoryl groups (proton-active, in XPS at around 132.2 eV). Instead, CXMs and CMXN exhibited a high pH_pzc_ value due to the amino groups on its surface (electron-active), granted by the modifying agent, melamine. Similar results were obtained by Moreno-Castilla [17].

**Figure 1:**
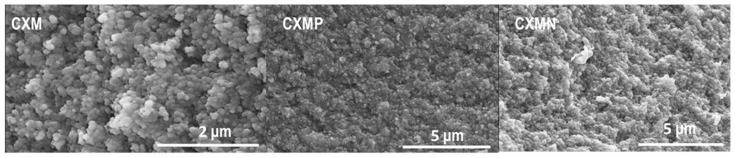
SEM micrographs for the internal structure of carbon xerogel microspheres (CXMs) and the CXMs functionalized with melamine (CXMN) and phosphoric acid (CXMP).

The morphology of CXM, CXMN, and CXMP samples was analyzed by SEM and representative micrographs of the internal structure are shown in Figure 1. All samples showed the characteristic structure of carbon gels, i.e., a three-dimensional network of agglomerated primary particles (0.0898 μm ± 0.006 μm) with inter particular spaces with pore size in the nanometric scale. The particle size distribution for CXM followed a unimodal Gaussian behavior. The results indicated that all samples have similar sizes of 12.3 μm ± 0.7 μm, as the functionalization process did not alter the size of the particle. In this sense, more textural properties of the materials such as surface area (S_BET_) and porous volume (micro-, meso- and macro-porous) are summarized in Table 2.

The heteroatoms inclusion in the surface of the CXM material decreased the porous spaces due to the blocking of the micro- and mesopores by loading the new oxygenated, nitrogenated, and phosphorated chemical groups [18]; this is related directly to the atomistic concentration obtained by XPS. The CXMP has an atomic concentration higher than the CXMN; indeed, the atomic size of nitrogen is lower than that of phosphorous, which favors the reduction of the S_BET_ due to the load and size of heteroatoms in the surface of the support. Finally, the macroporous space is not altered by the addition of heteroatoms (P and N). 

**Figure 2:**
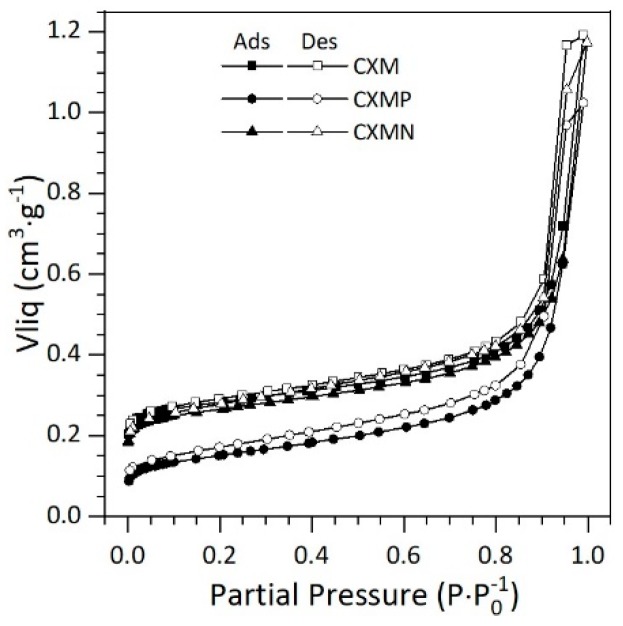
Nitrogen isotherms of carbon xerogel microspheres (CXMs) and CXMs functionalized with melamine (CXMN) and phosphoric acid (CXMP). Adsorption curve—filled symbols; desorption curve—unfilled symbols.

All samples can be defined as micro/mesoporous materials, with mesoporosity being the most remarkable distribution; i.e., Vmeso for CXMs, CXMP, and CXMP is between 71% and 85% of the total volume (VT). To better understand these results, the isotherms are presented in the Figure 2. 

All materials exhibited type IV isotherms. This isotherms type assumes monolayer adsorption at low pressures (micropores), as well as the formation of multimolecular layers at medium and high pression ranges (mesopores) until reaching a maximum multilayer thickness at a high Pressures. This isotherm is characteristic of mesoporous solids. Similar results have been previously reported by Bailón-García et al. [16] and Carrasco-Díaz et al. [19].

### 2.2. CBD Adsorption onto Carbon Xerogel Microspheres

**Figure 3:**
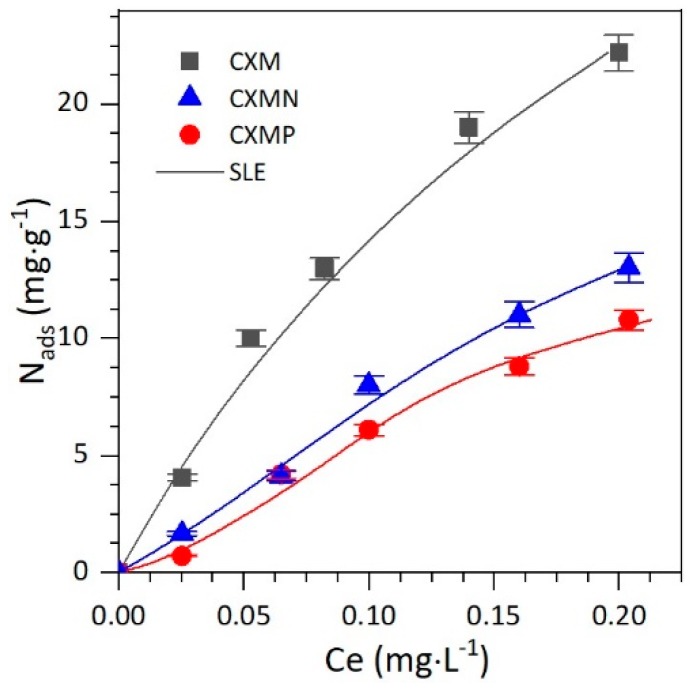
Adsorption isotherms at 25 °C of cannabidiol (CBD) in an aqueous solution of pH = 7 onto carbon xerogel microspheres (CXMs) and CXMs functionalized with melamine (CXMN) and phosphoric acid (CXMP), with experimental data fitted to the solid–liquid equilibrium (SLE) model.

The drug load onto the sample’s surface was done through batch-adsorption experiments at 25 °C and pH of 7.0 to favor the fluid (CBD solution)–solid (CXMs, CXMN, and CXMP) interactions. The adsorption experiments were carried out using different concentrations of CBD (<300 mg∙L^−1^) at a fixed CBD solution (mL) to an adsorbent ratio of 1:10 (see Section 3.2.4). Figure 3 shows the obtained adsorption isotherms for CBD onto each material. As observed in Figure 3, the isotherms of CBD adsorption obtained for the three materials are type I(b) according to the International Union of Pure Applied Chemistry (IUPAC), in which the amount adsorbed (Nads) increases as the equilibrium concentration (Ce) of CBD increases. The type I(b) isotherm indicates that the adsorbate could be adsorbed onto the adsorbent surface in a monolayer [19]. Besides, the adsorbed amount of CBD for the wide range concentration of concentrations evaluated showed a decrease in the order CXM > CXMN > CXMP. 

For the analysis of the adsorption isotherms the experimental data were fitted to the Solid-liquid equilibrium (SLE) model [20], which provides information about the affinity between the adsorbent and adsorbate (*H* parameter), and the degree of adsorbate self-association over the adsorbent surface (*K* parameter). The obtained parameters of the SLE model for each material are presented in Table 3. It can be observed in Table 3 that the adsorption isotherms have a good fit towards the SLE model, with root mean square error (RMSE%) <10%. The *H* parameter follows the order CXM > CXMN > CXMP, indicating that the material without functionalization has higher affinity for CBD adsorption. Also, the values of the *K* parameter suggest that there is a higher self-association for the functionalized materials than for the support.

It is worth mentioning that the adsorption isotherms of CBD for all samples were done at a pH of 7.0, and the points of zero charges (pH_pzc_) of CXMs, CXMN, and CXMP were 9, 7.7, and 2.7, respectively, as was reported in Table 1. In this sense, if pH_pzc_ is the pH value at which the total net charge on the surface of the adsorbent is neutral, the number of positive and negative sites is equal. Then, for pH values above pH_pzc_ negative sites are favored, and the material becomes affine to cationic molecules. Meanwhile, when the absorbent is exposed to pH values below pH_pzc_, the surface is loaded positively and is affine to anionic materials. In this study, the heteroatoms addition on the surface modified the pH_pzc_ value. Particularly, CXMP materials showed the lowest pH_pzc_, which implies that at pH = 7.0 the materials are negatively charged, producing a vigorous repulsion with the CBD having a partially negative charge (π–delocalized electron). Conversely, when CXMs and CXMN materials are exposed to the immobilization pH = 7.0, their surface charge density is positive, and attraction of the CBD is favored. These results are in accordnace with the results reported by Betancur et al. [21], who studied the adsorption of different types of surfactants onto silica-gel nanoparticles. 

### 2.3. Desorptin Kinetics of CBD from Carbon Xerogel Microspheres

**Figure 4:**
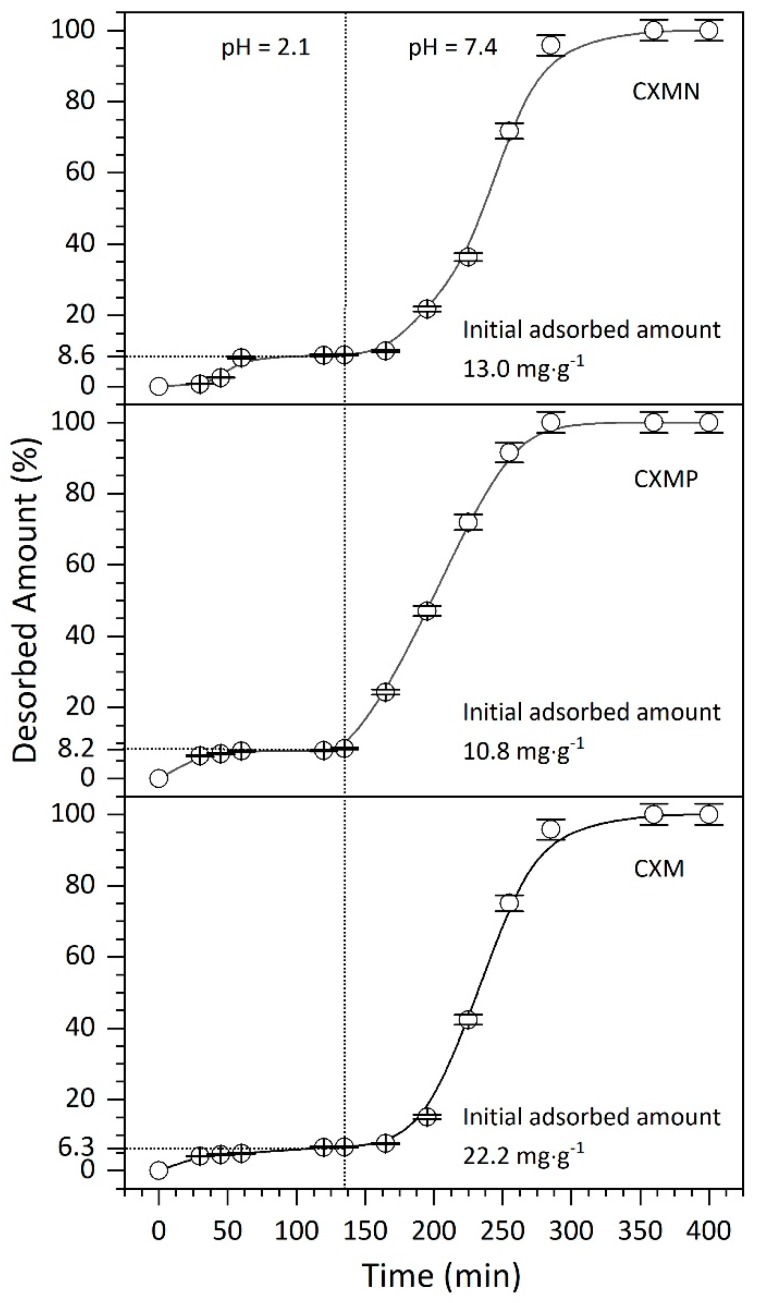
Desorption kinetics of cannabidiol (CBD) from carbon xerogel microspheres (CXMs) and CXMs functionalized with melamine (CXMN) and phosphoric acid (CXMP) at 37 °C and at pH = 2.1 (gastric medium: buffer CH_3_COONa/CH_3_COOH) and pH = 7.4 (final desorption at intestinal conditions: buffer Na_2_HPO_4_/NaH_2_PO_4_).

The desorption kinetics of CBD at 37 °C and pH = 2.1 (gastric medium: buffer CH_3_COONa/CH_3_COOH) and at pH = 7.4 (final desorption at intestinal conditions: buffer Na_2_HPO_4_/NaH_2_PO_4_) are shown in Figure 4. The results showed that at a pH = 2.1, the release of CBD was less than 9.0% with respect to the initial adsorbed amount, indicating an inefficient behavior of delivery for all the samples. The CBD desorption is inversely proportional to its load, i.e., CXM has the highest CBD load, but its desorption percent is the lowest, as can be seen in Figure 4. In conclusion, the desorption process is inefficient under gastric conditions, which is excellent for the main objective of this study. This behavior can be explained because all materials showed a positive surface under at pH = 2.1 (pH_pzc_ higher than 2.7), producing powerfully electrostatic attractions between the adsorptive couple (cation-delocalized π bonding) [22]. 

Subsequently, when the materials are exposed to intestinal conditions (pH = 7.4), the delayed release of residual CBD (>90%) which was not delivered in gastric conditions is favored. Under this condition, the attraction forces are reduced since the materials become less positive (weak cation–π bonding). Even for the CXMP sample with a pH_pzc_ of 2.7, the surface charges turn negative, leading to a high repulsion with the CBD molecule. This hypothesis can be validated with the desorption rates (DR) at 30 min after exposing all the materials to intestinal conditions, showing a DR for CXMP higher than for the other materials. The results obtained of DR were 0.14%∙min-1, 0.23%∙min-1, and 0.60%∙min-1 for CXMs, CXMN, and CXMP, respectively. There is evidence that for the oral administration of pure CBD the absorption is slow, erratic, and variable among individuals. Its oral bioavailability can vary between 5% and 10%, and the maximum plasma concentration (Cmax, ng∙mL^−1^) for a dose of 20 mg in men and 15 mg in women is 14.5 ng∙mL^−1^ ± 9.7 ng∙mL^−1^ and 9.4 ng∙mL^−1^ ± 4.5 ng∙mL^−1^, respectively [23]. The limitations are associated with the fact that the CBD is partially destroyed by the gastric juice and the low solubility in biological fluids [23]. Further, the results of the present study indicate that the proposed material guarantee the retention and maintenance of CBD under gastric conditions and its subsequent delayed-release in doses under intestinal conditions, which could lead to an increase in the bioavailability of CBD and, consequently, favor the oral administration of the compound. 

### 2.4. Aflatoxin B1 (AFLA_B1_) Removal with Carbon Xerogel Microspheres

**Figure 5:**
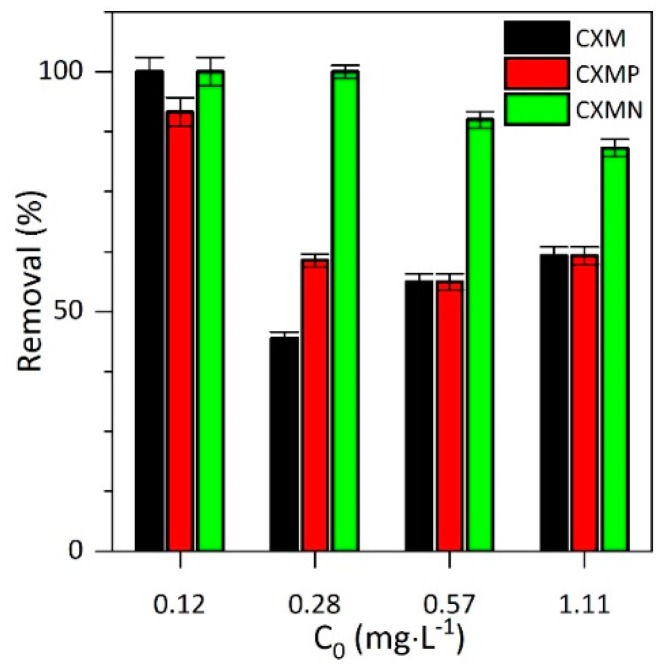
Aflatoxin B1 (AFLA_B1_) removal at 15 min from AFLA_B1_ solutions at concentrations between 0.1 mg∙L^−1^ and 10 mg∙L^−1^ in Na_2_HPO_4_/NaH_2_PO_4_ buffer at pH = 7.4 using carbon xerogel microspheres (CXMs) and CXMs functionalized with melamine (CXMN) and phosphoric acid (CXMP) at 37 °C.

The aflatoxin B1 (AFLA_B1_) is a toxic secondary metabolite produced by fungi [24]. When the conditions of humidity and temperature are adequate, fungi proliferate and form colonies that can produce high concentrations of AFLA_B1_. Cereals, nuts, and fruits are the places for the growth of fungi and the consequent production of AFLA_B1_ [24]. Also, because they are thermostable and resistant, they persist during the processing of food products, thus entering into the human food chain [25]. Exposure to high levels of AFLA_B1_ causes acute necrosis, cirrhosis, and liver cancer. In the long term, these compounds have carcinogenic, mutagenic, teratogenic, estrogenic, immunotoxic, nephrotoxic and neurotoxic effects. Several strategies have been developed to avoid the negative effects caused by mycotoxin consumption, for example, the use of adsorbent agents [26]. The present study demonstrated the dual ability of carbon xerogel microspheres as, after the CBD delayed delivery mainly at intestinal conditions, these materials can adsorb AFLA_B1_. Specifically, the materials assessed reached removals of aflatoxin between 40% and 100% in just 15 min after starting the test, as shown in Figure 5. The adsorption efficiencies are given in the following order CXMN > CXMs ≈ CXMP for the AFLA_B1_ concentration evaluated. Initially, it could be thought that the adsorption of AFLA is conditioned by the microporous space of the materials, in which the AFLA molecules can lodge. Hence, materials such as the CXMP with smaller micropore spacewould have the lowest removal rates under all concentrations evaluated. However, CXMN showed greater affinity for AFLA than its CXM analogue, even when it had a smaller surface area. This could be explained considering that although there is a slight reduction of the microporous space when nitrogen is added to the surface, this reduction is compensated by an increase in the affinity of nitrogen groups for the AFLA. The strong affinity between the adsorptive couple AFLA_B1_–CXMN compared to unmodified material is due to hydrogen bonds between electrons from AFLA_B1_ aromatics rings or free electrons on electronegative atoms from AFLA_B1_ and the hydrogens covalently bound to the nitrogen atom on the surface of CXMN [27]. Conversely, the pKa values for both molecules, CBD and AFLA_B1_, are greater than 9; therefore, this property does not play an important role in the interaction between the adsorptive couple in the pH range in which the adsorption/desorption phenomena occur.

**Figure 6:**
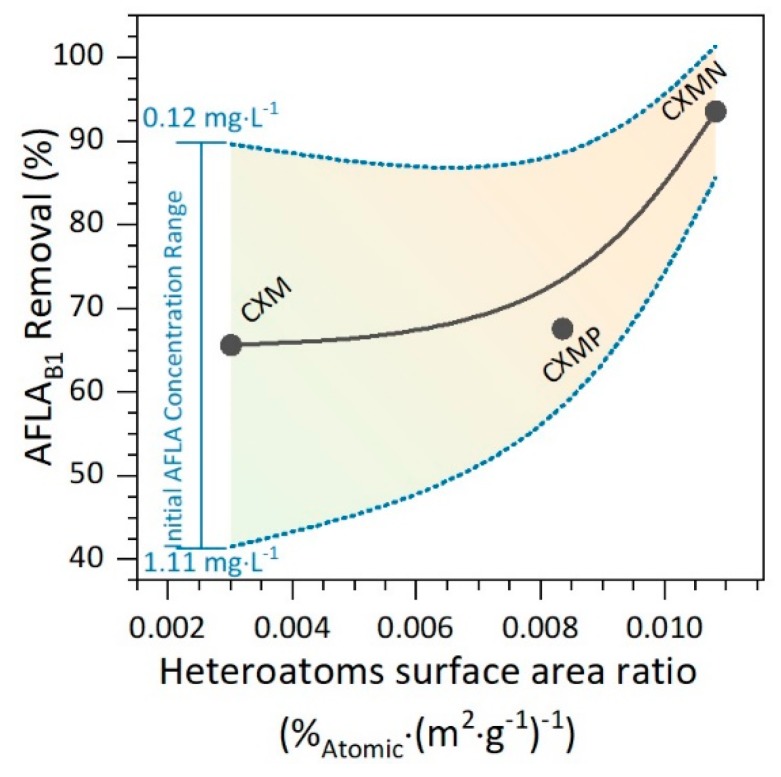
Aflatoxin B1 (AFLA_B1_) removal at 15 min as a function of heteroatoms per area unit (polarity), using carbon xerogel microspheres (CXMs) and CXMs functionalized with melamine (CXMN) and phosphoric acid (CXMP) at 37 °C.

On the other hand, materials without polar groups on the surface reduce their adsorptive capacity to the microporous space; hence, CXM exhibits massive falls in its ability to remove AFLA when its concentration increases in the medium, as a consequence of AFLA saturation of the active sites. In general, the adsorbate concentration had a negative effect on the removal efficiency, i.e., the higher the initial AFLA_B1_ concentration the lower is its removal. This makes sense, since the microporous spaces of the biomaterial decrease when the amount of adsorbate in the medium increases, generating a reduction in the total adsorptive capacity due to the active site saturation [22]. It can be seen from Figure 6 that as the number of heteroatoms per unit area (polarity) increases in the carbonaceous materials, the adsorptive capacity of the same becomes higher (AFLA_B1_ removal). 

The results of the present study are promising, since the World Health Organization (WHO) recommends the absence of AFLA_B1_ in organisms due to its carcinogenic, mutagenic, and teratogenic effects [27]. The limitations in the removal of aflatoxins are founded on the inability of conventional methodologies based on the use of adsorbents to eliminate concentrations of AFLA_B1_ in the order of the mg·L^−1^. However, in the present work the complete removal was reached in a period of less than 30 min using a low amount of the synthesized materials. 

## 3. Materials and Methods 

### 3.1. Materials 

Cannabidiol (CBD) solution in methanol at a concentration of 1 mg∙mL^−1^ was used as a stock solution for the calibration curve and adsorption experiments (Sigma-Aldrich, St. Louis, MO, USA). Resorcinol (≥90%, Sigma-Aldrich), formaldehyde (mass fraction of 36.5 in H_2_O, Sigma-Aldrich), and barium acetate (≥99%, Sigma-Aldrich) were used for the carbon xerogel microsphere (CXM) synthesis, while phosphoric acid (mass fraction of 85% in H2O, Sigma-Aldrich) and melamine (99%, Sigma Aldrich) were used for the material functionalization. Acetone (mass fraction of 99.8% in water, Sigma-Aldrich) was used for the washing protocol. Sodium acetate (99%, Merck KGaA, Darmstadt, DE, Germany), acetic acid (≥90%, Merck KGaA), sodium phosphate dibasic (≥90%, Sigma-Aldrich), and sodium dihydrogen phosphate (≥90%, Sigma-Aldrich) were used for preparing buffer solutions. Finally, standards of cannabidiol CBD (1 mg·mL-1, Sigma-Aldrich) and aflatoxin B1 (AFLA_B1_, 20 µg·mL^−1^ Sigma-Aldrich) were used for calibration curves and adsorption assays. 

### 3.2. Methods 

#### 3.2.1. Synthesis of Mesoporous Carbon Xerogels

Xerogels were prepared by polycondensation of resorcinol (R) with formaldehyde (F) in aqueous (W) media. A mixture of resorcinol, formaldehyde, and BaC_4_H_6_O_4_ catalyst in a molar ratio R:F:C equal to 1:2:0.001 was used; the mixture was put into glass molds (30-cm length and 0.5-cm internal diameter) and sealed. Molds were cured as follows: 24 h at 25 °C, 24 h at 50 °C, and finally 72 h at 80 °C. Posteriorly, these rods were cut into 2-cm pellets and placed in acetone for three days, to reduce the porosity collapse during the subsequent drying process. The pellets were dried by microwave heating using a Saivod MS-287 W (Spain) microwave oven under Argon atmosphere in periods of 1 min at 384 W until constant weight. Pyrolysis was carried out at 900 °C for 2 h in N_2_ flow (300 cm^3^∙min^−1^) to obtain the carbon xerogels microspheres (CXMs). The materials were kept in a desiccator until analysis [28].

#### 3.2.2. Functionalization of Carbon Xerogel Microspheres 

The surface of the CXM material was modified with phosphoric acid and melamine. The method applied for CXM functionalization was incipient impregnation [28], which consists in dissolving an amount of phosphoric acid or melamine in water and ethanol, respectively, with the objective of adding heteroatoms of phosphorous (CXMP) and nitrogen (CXMN) to modify the material’s surface chemical. Then, the solutions are slowly dropped onto the materials under constant homogenization. The impregnated materials were dried using an IR lamp for 24 h and later carbonized at 700 °C for 1 h with N_2_(g) as the inert atmosphere, finally the samples were washed with distilled water until constant pH to remove weakly adherent modifying agents. 

#### 3.2.3. Characterization of Carbon Xerogel Microspheres 

Materials morphology was studied by scanning electronic microscopy (SEM) using an LEO (Carl Zeiss, Germany) GEMINI-1530 microscope. The particle size (D_µm_) was calculated by SEM microphotographs analysis using the IJ.JAR software from Java ^®^. A minimum of 500 particles were studied from different photographs and their diameter calculated by the program assuming spherical shape. 

The textural characterization was carried out by N2 adsorption at −196 °C using a Quadrasorb SI equipment (Quantachrome instrument, Viena, Austria) [29]. The Brunauer–Emmett–Teller (BET) equation was applied to the N_2_-adsorption isotherms to determine the surface area (SBET) [29]. The Dubinin–Radushkevich equation was used to determine the micropore volume (Vmic). Mercury porosimetry was carried out using an AutoPore IV 9510 equipment (Micromeritics, Boynton Beach-Florida, USA) up to a pressure of 60.000 psi to obtain the volume of mesopores Vmeso (dpore 6 nm to 50 nm) and the macropore volume Vmacro (dpore 50 nm to 10,000 nm). The total pore volume (VT) was considered as Vmicro + Vmeso + Vmacro. [30]. The surface chemical characterization of the materials was analyzed by X-ray photoelectron spectroscopy (XPS) and the pH_pzc_ value according to the methodology previously described by [29]. 

#### 3.2.4. Adsorption of Cannabidiol (CBD) on Carbon Xerogel Microspheres 

Adsorption isotherms were carried out, adding a constant mass of each material to the CBD solutions at different concentrations, which were prepared based on the stock solution [23]. The ratio adsorbent mass to solution volume was of 1 g:100 mL The CBD solutions were previously prepared in sodium phosphate buffer of pH = 7.0 at concentrations between 20 mg∙L^−1^ and 300 mg∙L^−1^. Each sample containing the adsorbents was stirred at 100 rpm for 10 h at 25 °C to reach the sorption equilibrium. The amount of CBD adsorbed by the carbons in the equilibrium was calculated according to Equation (1):

(1) $$\mathrm{Nads}=\frac{(C_{0}-C_{e})\;V}{M}$$

where C_0_ and Ce are the initial and equilibrium CBD concentration, respectively (mg∙L^−1^), V is the solution volume (L), and M is the material mass (g). For the quantification of CBD, a calibration curve using cannabidiol (CBD) as standard and HPLC analysis was used, according to the protocol previously described by De Backer et al. [31]. 

#### 3.2.5. In-Vitro Release of CBD from Carbon Xerogel Microspheres

For CBD release tests, the load-drug materials obtained from the adsorption assays were added to buffer solutions simulating physiological conditions. Initially, CBD release at pH = 2.1 was evaluated (gastric medium: buffer CH_3_COONa/CH_3_COOH). Then, the composites were exposed at pH = 7.4 to assess the final desorption at intestinal conditions (buffer Na_2_HPO_4_/NaH_2_PO_4_), and the assays were performed at a fixed temperature of 37 °C. The amount of CBD desorbed by the carbons in the equilibrium was calculated according to Equation (2):

(2) $$\mathrm{Desorbed}\;\mathrm{amount}=\frac{C_{e}*V}{\mathrm{Nads}*M}$$

where Ce is the desorbed CBD concentration in the equilibrium (mg∙L^−1^), V is the solution volume (L), Nads is the maximum adsorbed amount (mg∙g^−1^), and M is the material mass (g).

#### 3.2.6. Adsorption of Aflatoxin B1 (AFLA_B1_) on Carbon Xerogel Microspheres 

After the desorption of CBD under intestinal conditions, the capacity of the materials to adsorb aflatoxin B1 was evaluated. For this, the materials were added to AFLAB1 solutions at concentrations between 0.1 mg∙L^−1^ and 1.1 mg∙L^−1^ in buffer Na_2_HPO_4_/NaH_2_PO_4_ at pH = 7.4, and the samples were stirred at 100 rpm for 15 min at 37 °C to reach the sorption equilibrium. The amount of AFLABB1 adsorbed by the materials in the equilibrium was calculated according to Equation (1) as described above. For the quantification of AFLA_B1_, a calibration curve using AFLA_B1_ Standard and HPLC analysis were used, according to the protocol previously described by Herzallah et al. [32].

#### 3.2.7. Statistical Analysis

The material characterization tests, as well as the adsorption and desorption assays, were made by triplicate (*n* = 3). The data reported corresponds to the arithmetic means. In neither case, deviations (± SD) exceeded 10% of the means (coefficient of variation). To determine statistical differences between measurements, unidirectional analysis of variance was performed using Statgraphics Centurion V (Statgraphics, Seattle-Washington, USA) based on the 95% confidence interval). Also, means comparisons were made through Duncan’s multiple range test (*p* < 0.05).

## 4. Conclusions

The present work evaluated the capacity of carbon xerogel microspheres with a different chemical nature to deliver cannabinol in gastric and intestinal conditions, as well as the simultaneous capacity for AFLAB1 removal. The results allow us to conclude that surface chemistry largely determines the affinity of the adsorptive couple; that is, positively charged materials such as CXMs and CXMN experience a strong attraction to the CBD molecule due to the type π–cation interactions. However, the desorption process was insufficient under gastric conditions due to the enhancing of opposite charges between the adsorptive couple at pH 2.1, which is opposed to intestinal conditions that weaken the attraction and allow the controlled release of CDB (pH = 7.4). Finally, the present work demonstrated the dual ability of microspheres (10 mg) to adsorb AFLAB1, showing removal values of 40% and 100% at initial concentrations of 0.1 and 1.1 mg∙L^−1^, respectively, after 15 min. This is the first study that involves the physicochemical versatility of carbon xerogel microspheres as dual-purpose materials for delayed release of CBD and AFLA_B1_ removal in simulated intestinal and gastric conditions to determine medicinal or co-adjuvant actions.

## Figures and Tables

**Table 1:** Zeta potential at zero charges (pH_pzc_) and atomic concentration on the surface of carbon xerogel microspheres (CXMs) and CXMs functionalized with melamine (CXMN) and phosphoric acid (CXMP).

Sample	Heteroatom Concentration (%)					pH_pzc_
	C1s	O1s	N1s	P2p	Heteroatoms (O+N+P)	
CXM	98.1	1.9	0	0	1.9	9.0
CXMN	97.1	1.8	1.1	0	2.9	7.7
CXMP	92.6	5.2	0	2.2	7.4	2.7

**Table 2:** Textural properties of surface area (S_BET_), and volume of micro-pores (Vmicro), mesopores (Vmeso), macro-pores (Vmacro), and total pore volume (Vtotal) for carbon xerogel microspheres (CXMs) and CXMs functionalized with melamine (CXMN) and phosphoric acid (CXMP).

Sample	S_BET_	Vmicro	Vmeso	Vmacro	Vtotal	Vmicro	Vmeso	Vmacro
		(0.5–2.0) nm	(6.5–50) nm	(50–10000) nm	(0.5–10000) nm	(0.5–2.0) nm	(6.5–50) nm	(50–10000) nm
	(m^2^∙g^−1^)	cm^3^∙g^−1^			cm^3^∙g^−1^	%		
CXMs	683	0.243	0.909	0.03	1.186	20	77	3
CXMN	638	0.227	0.658	0.04	0.929	24	71	5
CXMP	346	0.123	0.825	0.04	0.989	12	83	4

**Table 3:** Parameters obtained for the solid–liquid equilibrium (SLE) model from adsorption isotherms of cannabidiol (CBD) in an aqueous solution of pH = 7 onto carbon xerogel microspheres (CXMs) and CXMs functionalized with melamine (CXMN) and phosphoric acid (CXMP) at 25 °C. The *H* parameter is related to the adsorption affinity, the *K* parameter is related to the adsorbate self-association over the adsorbent surface, and *Q_max_* is the maximum amount adsorbed. RMSE: root mean square error.

Material	*H* (mg/g)	*K* (g/g)	*Q_max_* (g/g)	RMSE%
**CXM**	5.13	0.47	0.099	9.8
**CXMN**	18.26	97.47	0.022	5.6
**CXMP**	48.60	422.91	0.013	8.4
